# The Bolingbroke Hospital

**Published:** 1906-05-12

**Authors:** 


### THE BOLINGBROKE HOSPITAL.

On Saturday, May 5, the Princess Royal, accompanied by the Duke of Fife, visited the Bolingbroke Hospital for the purpose of laying the foundation-stone of the new building. A large concourse of people awaited the arrival of the Royal party in the pavilion erected on the site of the new building. The Princess and the Duke of Fife arrived punctually at 3.30, and, having been met by the Reception Committee, were conducted to the pavilion. The Bishop of Kingston then offered up some dedicatory prayers, after which Canon Erskine Clarke, President and Chairman of the hospital, gave a short address, in which he briefly sketched the past history of the hospital up to the time of its incorporation as a "Free Accident Hospital" in 1897. Since that time its work had greatly increased, and although it still provided private wards for paying patients, yet the majority of cases were those who had been admitted from accident or emergency. In 1901 an out-patient department, the first section of a general hospital of 153 beds, was built at a cost of £6,000 as a memorial to the late Queen Victoria. This department was of great value to the district, and every year treated thousands of cases; the number treated last year exceeded 27,000. The number of in-patients in 1905 was 592. The Committee of King Edward's Hospital Fund had for several years urged the governors to build a further portion of the planned hospital, and had given such substantial help that the governors had now sanctioned an expenditure of £30,000 —no small venture of faith in such a district, and at such a time as the present. It was to further this effort that the presence of the Princess had been solicited, and in the name of the governors, the staff, and the friends of the hospital, he offered their grateful appreciation of Her Royal Highness's condescension in thus coming with his Grace the Duke of Fife to their aid.

The Duke of Fife replied that he had been asked by the Princess Royal to thank them for the kind expressions contained in the address which had just been read, and to assure them that it gave her very great pleasure to inaugurate so important an undertaking. It gave him also very great pleasure to accompany the Princess, and they were both thoroughly alive to the importance, indeed, the necessity, of providing hospital accommodation in this district, where the growth of the population during the last quarter of a century had been phenomenal. To all those who, like themselves, took an interest in hospital work, the history of the Bolingbroke Hospital was extremely interesting, because it had been founded upon the wisest of all wise principles—the paying principle. Although he gathered that the hospital had now been converted into a general hospital, he wished to express the hope that that wise principle—the paying principle—would not be entirely discarded in its administration, because he was convinced that if that system had been more universally adopted the financial difficulties which hampered all the hospitals would not be so serious. It was not an opportune moment for making a long speech, and he would only, in a few words, offer, on behalf of his wife and himself, their hearty congratulations to the inhabitants of that neighbourhood on having overcome the initial difficulties with regard to the enlargement of that institution. They both earnestly hoped that the difficulty of maintenance might be met and overcome by the generosity and public spirit of the inhabitants of that wide and thickly-populated neighbourhood, if only the thousands of people who lived within hail of the Bolingbroke Hospital would combine to assist to keep it up according to their means, some with pence, some with shillings, and others with pounds. Their best wishes for the success of this much-needed institution would, he was sure, be realised, but only by combination could this or any other institution be kept up.

Her Royal Highness then laid the foundation-stone, and subsequently purses for the building fund were presented, among which was one collected among the employés of Price's Candle Factory, amounting to £40 10s. A purse presented by Mrs. Dudley Bennett contained £35, and had been collected in penny subscriptions from working men at the Shakespeare Theatre. Seventeen purses were presented containing £100 altogether. Miss T. Russell, the matron, presented a very pretty bouquet of pink roses. The Royal party then took their departure, and the guests were afterwards served with tea, the pipers of the Royal Caledonian Asylum playing the while.

Subscriptions towards the building fund were still coming in, a donation of £500 having just been received from Lord Ashcombe.

